# Genetic sequencing of children with malrotation and midgut volvulus: A cross‐sectional study

**DOI:** 10.1002/jpr3.70230

**Published:** 2026-08-14

**Authors:** Jonathan A. Salazar, Shira Rockowitz, Courtney E. French, Wanqing Shao, Daniel M. Elman, Alexandra N. Carey, Tom Jaksic, Piotr Sliz, Wen Hann Tan, Christopher P. Duggan

**Affiliations:** ^1^ Division of Gastroenterology, Hepatology, and Nutrition Connecticut Children's Hartford Connecticut USA; ^2^ Connecticut Children's Intestinal Rehabilitation Program Connecticut Children's Hartford Connecticut USA; ^3^ Children's Rare Disease Collaborative Boston Children's Hospital Boston Massachusetts USA; ^4^ Division of Genetics and Genomics Boston Children's Hospital Boston Massachusetts USA; ^5^ The Manton Center for Orphan Disease Research Boston Children's Hospital Boston Massachusetts USA; ^6^ Department of Pediatrics Boston Children's Hospital Boston Massachusetts USA; ^7^ The Institutional Centers for Clinical and Translational Research Boston Children's Hospital Boston Massachusetts USA; ^8^ Division of Gastroenterology, Hepatology, and Nutrition Boston Children's Hospital Boston Massachusetts USA; ^9^ Center for Advanced Intestinal Rehabilitation Boston Children's Hospital Boston Massachusetts USA; ^10^ Department of Surgery Boston Children's Hospital Boston Massachusetts USA; ^11^ Division of Molecular Medicine Boston Children's Hospital Boston Massachusetts USA; ^12^ Department of Biological Chemistry and Molecular Pharmacology Harvard Medical School Boston Massachusetts USA

**Keywords:** intestinal failure, parenteral nutrition, short bowel syndrome

## Abstract

**Objectives:**

Intestinal malrotation with midgut volvulus can cause a particularly severe form of pediatric intestinal failure and is often a cause of ultra‐short bowel syndrome (SBS), with longer dependence on parenteral nutrition. While malrotation can be found in several genetic syndromes, most occurrences of this condition are not associated with any identifiable genetic conditions or syndromes. This study aimed at identifying specific genes associated with this phenotype in humans.

**Methods:**

Children with a history of SBS due to malrotation with midgut volvulus were identified. Children with SBS due to multiple diagnoses, with non‐midgut volvulus, or with an identified genetic syndrome were excluded. Whole‐genome sequencing (WGS) was performed on the proband and their biological parents.

**Results:**

Twenty‐three families provided consent to be enrolled in the study, and WGS was obtained from 21 complete families (proband and two parents). Two patients had variants in the glutathione S‐transferase theta 4 gene (*GSTT4*). Two patients had variants in the coiled‐coil domain‐containing 175 gene (*CCDC175*). Two patients had a de novo heterozygous variant in the WW domain‐containing binding protein 4 gene (*WBP4*). Three patients had de novo heterozygous likely pathogenic variants in *TFRC*, which encodes the transferrin receptor.

**Conclusions:**

We identified four genes of potential interest in the etiology of malrotation and midgut volvulus, suggesting that the condition may be genetically heterogeneous. While the genes of interest identified have not yet been shown to be involved in the pathophysiology of this condition, they provide important areas for further investigation.

## Abstract (highlights)

What is Known

- Intestinal malrotation with midgut volvulus is a severe form of short bowel syndrome.
- Certain genetic syndromes are associated with this diagnosis and can be identified early in life.
- A high proportion of cases of midgut volvulus with intestinal malrotation occur in the absence of any identifiable genetic predisposition.

What is New

- Whole‐genome sequencing was performed on patients with intestinal malrotation and midgut volvulus without known underlying genetic or syndromic cause.
- Four genes were identified to be potentially associated with intestinal malrotation and midgut volvulus.

## 1 INTRODUCTION

Pediatric intestinal failure (IF), defined as “the reduction of functional gut mass below the minimal amount necessary for digestion and absorption adequate to satisfy the nutrient and fluid requirements for growth,”^1^ is a complex but survivable syndrome.^2^ A particularly severe form of IF is intestinal malrotation (an arrest of the normal rotation of the embryonic gut) with midgut volvulus (a twisting of the mal‐rotated intestine around the mesentery with subsequent intestinal ischemia). The effects of ischemia can be limited with early surgical intervention, but in some cases, intestinal necrosis of large vascular territories of the superior mesenteric artery results in massive bowel necrosis and the need for resection.^3^ Notably, midgut volvulus is one of the more common causes of ultra‐short bowel syndrome (SBS), characterized by 10–25 cm of remnant small bowel,^4^ often leading to a longer duration of parenteral support. The extensive dependence on parenteral nutrition (PN) in these patients can lead to increased morbidity and possibly mortality. Hong et al. described an average of 2 years of PN dependence prior to achieving enteral autonomy and a high (78%) occurrence of small bowel bacterial overgrowth (SBBO) in these children.^5^ In addition, the literature has identified midgut volvulus as a risk factor for advanced intestinal failure‐associated liver disease (IFALD)^4^ as well as d‐lactic acidosis,^6^ two serious morbidities of IF.

Intestinal malrotation, which predisposes patients to midgut volvulus, has been noted to be a component of several other congenital syndromes, some of which have been identified to have genetic etiologies. These include megacystis microcolon and intestinal hypoperistalsis syndrome (MMIHS) (which may be associated with pathogenic variants in *ACTG2, MYH11, MYLK*, *LMOD1*, or *MYL9* genes),^7^ congenital SBS (associated with pathogenic variants in *CLMP*),^8^ Martinez–Frias syndrome (genetic etiology unknown), heterotaxia due to various etiologies,^9^ and a syndrome characterized by alveolar capillary dysplasia with misalignment of pulmonary veins.^10^ Martin et al. reviewed multiple examples of syndromic intestinal malrotation with a proven or possible genetic etiology, including abnormalities due to left and right patterning as well as diagnoses associated with other congenital malformations.^11^

Isolated intestinal malrotation, however, is a condition where abnormal rotation of the midgut occurs in utero in the absence of other organ malformations or functional deficits that would be suggestive of an underlying genetic syndrome. In fact, most patients with intestinal malrotation and midgut volvulus do not have any identifiable underlying genetic conditions nor many medical comorbidities, outside of the sequelae of the intestinal resection and its treatment. The genetic causes of isolated intestinal malrotation remain poorly understood. A recent study attempted to identify rare copy number variants (CNVs) in patients with intestinal malrotation without midgut volvulus. CNVs are differences in the number of copies of a specific segment of DNA in an individual's genome. These variations can arise from insertions, duplications, and deletions. Six rare CNVs were identified in five patients. Five CNVs involved previously identified syndrome loci, and one CNV of unknown significance (*GALNT14*) was found.^12^ *GALNT14* has not previously been associated with human disease but could serve as a potential candidate for further study due to its potential role in cell morphogenesis and organization of the dorsal mesentery during embryologic development.^13^ Isolated malrotation without midgut volvulus can occur, but this is not screened for and is often neither clinically significant nor apparent until a midgut volvulus occurs. Given the increasing understanding of the potential influence of genetics on the likelihood of developing isolated intestinal malrotation and subsequent risk of midgut volvulus, we conducted genome sequencing of children with pediatric IF secondary to SBS due to isolated malrotation with midgut volvulus, without an underlying known syndrome or genetic diagnosis.

## 2 METHODS

### 2.1 Ethics statement

Boston Children's Research Institutional Review Board (IRB) approval was obtained prior to initiation (study ID: IRB‐P00039894).

### 2.2 Study design

After IRB approval (IRB‐P00039894), we screened for potential candidates by conducting a medical record review of patients followed by the Center for Advanced Intestinal Rehabilitation (CAIR) at Boston Children's Hospital with the International Classifcation of Diseases (ICD‐10) Code for volvulus (K56.2) between January 1, 2015, and January 1, 2023. Between January 1, 2023, and April 1, 2024, additional candidates were identified by reviewing the weekly outpatient and inpatient schedules of the CAIR program. Subsequently, a review of the medical records was conducted, including operative reports, radiologic imaging, and clinic documentation to confirm the diagnosis of midgut volvulus in the setting of malrotation. Exclusion criteria were (1) diagnoses leading to SBS in addition to midgut volvulus (e.g., gastroschisis); (2) non‐midgut volvulus (i.e., segmental volvulus, sigmoid volvulus, intrauterine volvulus); and (3) any underlying identified genetic syndrome. The research team then coordinated with members of the CAIR Program to identify patients whose biologic parents were involved in the patient's lives.

### 2.3 Sample collection

Written informed consent was obtained from both parents, and assent was obtained when the child was 12 years or older. EDTA samples for genomic sequencing were obtained through the Experimental Therapeutics Unit at Boston Children's Hospital.

### 2.4 Genetic sequencing

Whole‐genome sequencing (WGS) for the Children's Rare Disease Collaborative (CRDC) was performed by GeneDx, a CLIA‐certified testing facility. Although sequencing was conducted on a research basis, additional DNA was stored for potential clinical confirmation. WES DNA was extracted using IDT xGen probes for samples prepared before 2024 and Twist v2.0.2 probes for samples prepared thereafter. The average WES coverage was 100×, with over 95% of target regions covered at ≥20× and mean target coverage ≥50×. WGS library preparation was performed using Illumina DNA PCR‐Free Prep Tagmentation, and sequencing achieved an average depth of coverage of at least 40×.^13^

### 2.5 Data processing

All genomic data were aligned to the reference genome build GRCh38 with the DRAGEN secondary analysis platform from Illumina,^14^ with v3.9 applied to data received prior to April 2024 and v4.2 used thereafter. Single‐nucleotide variants (SNVs), small insertions/deletions (indels), CNVs, structural variants (SVs), and disease‐associated short tandem repeat (STR) expansions were identified via DRAGEN. Mitochondrial genome variants were detected using Mutect2,^15^ and mobile element insertions were identified via xTEA.^16^ The resulting variant calls were annotated and made available to researchers through a combination of commercial and in‐house analysis platforms, including a GeneDx analysis platform, Emedgene (Illumina), and seqr.^17^

### 2.6 Statistical analysis

Using either the GeneDx research platform or seqr, we analyzed the variants identified using both a de novo dominant and an autosomal recessive model of inheritance because none of the participants had a known family history of midgut volvulus, and none of the parents were known to be affected. The variants were classified based on the American College of Medical Genetics (ACMG) guidelines.^18^ In the de novo dominant model, a maximum allele frequency of 0.001 was used as the threshold for filtering variants, whereas in the autosomal recessive model, a maximum allele frequency of 0.01 was used for filtering variants.^18^ Where available, we used the classification of pathogenicity provided by ClinGen (https://clinicalgenome.org/).

## 3 RESULTS

**Figure 1:**
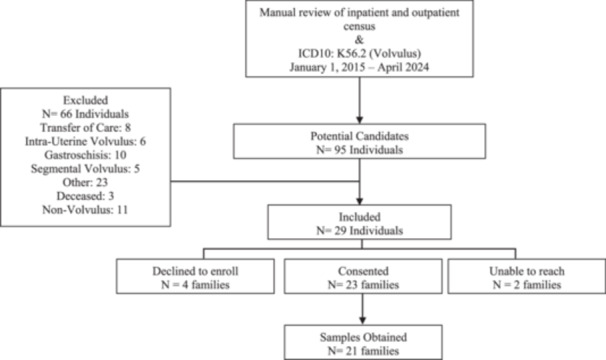
Study design and patient selection.

A total of 73 patients were identified on the initial review utilizing ICD‐10 codes, with 22 additional individuals identified through weekly review of the CAIR program census. Of 95 patients identified on initial medical record review, 29 met inclusion criteria (Figure 1). Of the 29 families approached, 23 provided consent to be enrolled in the study. Due to logistic limitations, genomic data were ultimately obtained from 21 patients (and their biological parents) and used in this analysis (Figure 1).

Baseline characteristics of the patients enrolled are presented in Table 1. Nearly half of all participants were diagnosed with malrotation in the neonatal period (<30 days of age). Five (23.8%) individuals were identified as female (XX) through genomic analysis. Mean (standard deviation [SD]) residual small bowel length at diagnosis was 42.3 (35.8) cm, and 80% had 70 cm or less. At the time of blood draw, about 33% of the patients were enterally autonomous with no PN dependence, 33% had less than 50% of calories from PN, and the remaining 33% had greater than 50% of calories provided for by PN. The cohort was generally well‐nourished, with a mean (SD) body mass index *Z* score of 0.04 (0.77).

**Table 1:** Descriptive statistics in 21 children with intestinal failure due to malrotation with midgut volvulus.

Variable	*n* (%) or mean (SD)
Age at diagnosis (months)	19.3 (26.3)
Age range (months)	1 month to 72 months
Birth to 1 month	10 (47.6%)
2–12 months	3 (14.3%)
12+ months	8 (38.1%)
Female sex (XX)	5 (23.8%)
Residual small bowel length at diagnosis	
0–35 cm	9 (42.9%)
35–70 cm	8 (38.1%)
>70 cm	4 (19.0%)
Calories from parenteral nutrition^a^^,^ b	39.2 (35.4)
No parenteral nutrition	7 (33.3)
1%–50%	7 (33.3)
>50%	7 (33.3)

Abbreviation: SD, standard deviation.

^a^ Values obtained at the time of laboratory draw.

^b^ Percentage of calories provided by parenteral nutrition is calculated as a fraction of the total provision of calories from both parenteral and enteral nutrition combined.

Two patients had variants in the Glutathione S‐transferase theta 4 gene (*GSTT4*); one patient had two heterozygous variants that were not paternally inherited; the other patient had a de novo heterozygous variant. Two patients had variants in the Coiled‐Coil Domain Containing 175 gene (*CCDC175*). One patient had a de novo heterozygous variant, and the other patient had a paternally inherited variant and a variant that was not paternally inherited. Neither *GSTT4* nor *CCDC175* is known to be associated with any human condition, so both are currently classified as “genes of uncertain significance.” Two patients had a de novo heterozygous variant in the WW domain‐containing binding protein 4 gene (*WBP4*). One patient's variant was of uncertain significance, and the other patient's variant was predicted to be likely pathogenic. In addition, three patients had de novo heterozygous likely pathogenic variants in *TFRC*, which encodes the transferrin receptor. Notably, one individual was incidentally noted to have a homozygous defect in the MYORG gene, which has been associated with primary familial brain calcification (PFBC). This neurodegenerative condition is characterized by varying degrees of dysarthria, cerebellar syndrome, gait anomalies, and pyramidal signs.^19^

## 4 DISCUSSION

While several syndromes have been reported to be associated with intestinal malrotation, no genetic causes have been identified for isolated intestinal malrotation with midgut volvulus.^11^ Our study aimed to identify possible genetic causes of this devastating condition in a cohort of pediatric patients with IF due to midgut volvulus, in the absence of any underlying syndrome or genetic diagnosis.

We identified four genes of potential interest in the etiology of isolated intestinal malrotation and midgut volvulus, suggesting that the condition may be genetically heterogeneous. These included *GSST*, *CCDC175*, *WBP4*, and *TFRC*. *GSST*, with a population carrier frequency of 1:3204, is thought to be involved in the glutathione metabolic process predominantly expressed in the testes^20^ but has not been associated with any human disease. *CCDC175* is a protein‐coding gene with ubiquitous expression in more than 25 tissues, including the testes, and has been associated with brain small vessel disease and cone‐rod dystrophy Type 2. Neither of these diagnoses is known to be associated with intestinal malrotation.^21^ Notably, however, the male‐to‐female ratio for intestinal malrotation has consistently shown a male predominance of 2:1.^22^ It remains unclear whether the expression of CCDC175 and GSST in the testes is associated with this predominance. *WBP4* encodes a spliceosomal protein. Biallelic variants in *WBP4* have been reported to result in a neurodevelopmental disorder with hypotonia, feeding difficulties, facial dysmorphism, and brain abnormalities; however, it is unknown whether some heterozygous carriers may present with intestinal malrotation.^23^, ^24^ Lastly, *TFRC* has a population carrier frequency of 1:4388 and encodes the transferrin receptor. Biallelic pathogenic variants in *TFRC* result in a form of primary combined immunodeficiency, but it is unknown whether carriers may have an increased risk for intestinal malrotation.^25^

There have been multiple other attempts to elucidate genetic causes of other forms of IF. The most common cause of IF in children remains necrotizing enterocolitis (NEC). The pathophysiology of NEC is multifactorial and includes differences in the immune system and inflammatory response, the microbiome, and the microcirculation. Cai et al. reviewed newer data that showed notable gene‐level differences in patients with NEC. For example, epigenetic modification through cytosine‐guanine dinucleotides (CpGs) methylation was discovered in genes involved in microvasculature and intestinal perfusion (VEGFA and DEFA5) as well as in genes associated with pro‐inflammatory pathways (IL‐17, CD40, and TLR4).^26^ Similarly, work done to elucidate the genetic causes of gastroschisis showed that this diagnosis is likely due to genetic‐environmental factors and was able to identify several genes of interest, including the gene coding for an adhesion receptor (ICAM1) that may play a role in this complex pathophysiology.^27^ Intestinal atresia is another cause of IF, and efforts have been made to identify genetic causes for these anomalies as well. Notably, tetratricopeptide repeat domain 7A (TTC7A) has been identified as the “steward of intestinal health,” and several mutations in this gene have been identified to be associated with intestinal atresia, very early onset inflammatory bowel disease (veo‐IBD), immunodeficiency, and changes in the intestinal epithelial architecture and integrity.^28^, ^29^

During the consenting process, the research team disclosed that a risk associated with genetic testing includes incidental findings with potential medical significance. Participants were able to opt in to be informed of all genetic findings or only those associated with their primary diagnosis. Notably, one individual was found to have a potentially life‐altering genetic diagnosis that was not related to their SBS. This family had made the decision to be made aware of all possible genetic findings. The research team communicated these results and facilitated further discussions and follow‐up with a genetics counselor.^30^

This study had some important strengths and limitations. To our knowledge, this is the first study to attempt to evaluate the genetic etiology of midgut volvulus in patients without an identifiable syndrome. The study was able to include children with a wide range of IF severity with regard to their remnant small bowel and degree of PN dependence. Study limitations include a small sample size due to the rare nature of this disease. As with other complex causes of IF, there is a strong likelihood that intestinal malrotation with midgut volvulus is a genetically heterogeneous condition, reducing the ability to identify a causative factor common to all families in the cohort.

## 5 CONCLUSION

From a genetic standpoint, conditions such as isolated intestinal malrotation can be (i) multifactorial, with a combination of genetic and intrauterine environmental factors; (ii) polygenic, with variants in multiple genes resulting in a condition due to additive or cumulative effects of the various mutations; or (iii) variants in single genes associated with a yet‐to‐be‐identified biologic process or condition. Regardless of the ultimate mechanism, the genes of interest identified have not yet been shown to be involved in the pathophysiology of isolated intestinal malrotation, but they provide important areas of interest for further investigation.

## CONFLICT OF INTEREST STATEMENT

The authors declare no conflict of interest.
